# Adaptability and Stability of Safflower Genotypes for Oil Production

**DOI:** 10.3390/plants11050708

**Published:** 2022-03-07

**Authors:** Sebastião Soares de Oliveira Neto, Douglas Mariani Zeffa, Gustavo Henrique Freiria, Tiago Zoz, Carlos Jorge da Silva, Maurício Dutra Zanotto, Renato Lustosa Sobrinho, Saud A. Alamri, Mohammad K. Okla, Hamada AbdElgawad

**Affiliations:** 1 Department of Crop Science, School of Agricultural Science, São Paulo State University, Botucatu 18610-034, SP, Brazil; zanotto@fca.unesp.br; 2 Department of Agronomy, State University of Maringá, Maringá 87020-900, PR, Brazil; douglas.mz@hotmail.com; 3 Department of Agricultural and Natural Sciences, Minas Gerais State University, Unidade de Ituiutaba, Ituiutaba 38302-192, MG, Brazil; gustavo.freiria@uemg.br; 4 Research Group for Innovation and Advancement of Agriculture, Mato Grosso do Sul State University, Unidade de Mundo Novo, Mundo Novo 79980-000, MS, Brazil; tiago_zoz@hotmail.com; 5 Instituto Federal de Educação, Ciência e Tecnologia de Mato Grosso, Campus Campo Novo do Parecis, Campo Novo do Parecis 78360-000, MT, Brazil; carlos.silva@cnp.ifmt.edu.br; 6 Department of Agronomy, Federal University of Technology, Pato Branco 85503-390, PR, Brazil; rsobrinho@alunos.utfpr.edu.br; 7 Botany and Microbiology Department, College of Science, King Saud University, P.O. Box 2455, Riyadh 11451, Saudi Arabia; salami@kste.edu (S.A.A.); mkkla@kste.edu (M.K.O.); 8 Integrated Molecular Plant Physiology Research, Department of Biology, University of Antwerp, 2020 Antwerpen, Belgium; hamada.abdelgawad@uantwerpen.be

**Keywords:** *Carthamus tinctorius* L., oilseed breeding, mixed models, parametric/non-parametric measures, Cerrado crops, biodiversity, agroecosystem diversification

## Abstract

The study aimed to analyze the agronomic performance of 11 safflower genotypes using adaptability and stability methods, while identifying safflower genotypes with stable behavior and a high grain yield in different environments of the Brazilian Cerrado. Ten lines and a cultivar of safflower were evaluated in four environments in the Brazilian conditions. Our results revealed the genotypes P30, P35, P9, P11, and P31 to be superior for grain yield and P43, P7, P11, and P31 to be superior for oil content. The lowest Wricke index, an indication of genotype stability, was observed for P9 (0.41%), which is considered the most stable genotype, followed by P35 (1.29%) and P31 (1.98%). For the predictability of the behavior of genotypes in the environments, P7 (80.85%), P35 (86.10%), P31 (85.90%), and P9 (97.42%) were considered predictable genotypes. The genotypes P11 (1045.6 kg ha^−1^ and 19.7%) and P21 (952.7 kg ha^−1^ and 20.6%) are recommended for cultivation in this region, considering both their grain yield and oil content. Safflower is viable to use out of season in the Brazilian Cerrado. The crop can generate profits for farmers and be used for oil production in periods of uncertain corn production.

## 1. Introduction

Safflower (*Carthamus tinctorius* L.) is a diploid and autogamous species [1], ranking eighth in the world among the most essential oilseeds [2]. Safflower oil has a wide range of uses, which can positively influence its yield, such as the production of pharmaceutical and culinary compounds [3] and biodiesel [4]. It is mainly used as an oilseed, although it can be used for other purposes due to its yellow-colored pigments.

Brazil does not have a significant safflower production; however, the species has shown great suitability for the country [4,5] mainly due to its drought tolerance traits [6], which allow its cultivation in regions that experience dry spells, such as in the Brazilian Cerrado, or that do not have an ideal rainfall for other crops, as in the northeast.

According to Singh and Nimbkar [7], the main objective of safflower breeding has been the development of genotypes with a high grain yield, high oil content, and higher resistance to diseases and pests. For this, several researchers in different regions and climates around the world developed studies on the inheritance of some agronomic traits and the combined ability of safflower genotypes [8,9].

The greater acceptability and use of safflower as an oilseed depend on breeding for the traits of interest and developing cultivars adapted to different growing regions [5,7]. Several studies have been carried out in Iran [8,10] and Brazil [5] to obtain safflower genotypes adapted to the contrasting environments. Aside from the commercial release of new cultivars, it is necessary to study the performance of several genotypes in different growing regions to understand the genotype × environment interaction. This interaction may lead to an inconsistency in classifying genotypes in the several environments tested [11,12]. Cruz et al. [13] emphasize that to reduce the effects of G × E interaction, it is necessary to evaluate the adaptability and stability of each genotype to find those with predictable behavior that respond to environmental variations under specific and general conditions.

Several methodologies have been reported for studying adaptability and stability in multi-environment trials. The methods proposed may be based on the components of an analysis of variance, the regression method, non-parametric methods, multivariate methods, mixed models, and recent methods based on factor analytic models [14,15,16,17,18,19].

Using methods to assess adaptability and stability to improve selection and an adequate indication of genotypes are essential for developing the safflower crop. There is a lack of information about safflower breeding in the literature, mainly with results derived from growing conditions in Cerrado. The region is promising for safflower cultivation since it has characteristics suitable for its growth, and there is no competition for the area with other crops. Therefore, studies on the adaptability and stability of safflower genotypes in this region are essential to indicate the best genotypes for cultivation. Based on this, this study aimed to analyze the agronomic performance of 11 safflower genotypes using adaptability and stability methods and to identify safflower genotypes with stable behavior and a high grain yield in different environments of the Brazilian Cerrado.

## 2. Materials and Methods

### 2.1. Genetic Material, Experimental Design, and Traits Assessed

Ten advanced lines of safflower (P43, P30, P28, P7, P35, P9, P11, P21, P31, and P14), selected in the Plant Breeding Program of the School of Agricultural Science (FCA), São Paulo State University (UNESP), and one control (cultivar IMA7326) were evaluated in four environments: two environments in the 2018 growing season—Bauru-SP and Botucatu-SP(1)—where sowing was carried out in March 2018; and two environments in the 2019 growing season—Botucatu-SP(2) and Campo Novo do Parecis-MT—where sowing was carried out in March 2019. The four environments were located in the Brazilian Cerrado. Details of the experimental location are shown in Table 1. The advanced lines of safflower were obtained through a selection based on grain yield and oil content. The segregating population conduction was performed by the single seed descent (SSD) method under field conditions. The base population was obtained in a genetic design in the diallel performed in previous years.

The experiments were arranged in a randomized block design with three replications. The plots were composed of four rows of 4 m length, 0.5 m of row spacing, and a density of 10 plants per meter. In all experiments, the fertilization with 200 kg ha^−1^ of an NPK formulation (08-28-16) was carried out according to the soil analysis. Weeds were controlled with manual weeding, and no chemical control of pests and diseases was necessary.

The average grain yield (GY), given in kg ha^−1^, was determined for each genotype in each environment by harvesting and processing the plants in the two central rows of the plots. The oil content (%O), given as a percentage, was obtained through the average of three readings of a predetermined volume of seeds, carried out for each plot, using a magnetic resonance spectrometer, model SLK-100 (SpinLock, Cordoba, Argentina).

### 2.2. Statistical Analyses

Initially, individual analysis of variance was performed to verify the magnitudes of the residual mean squares. Then, a joint analysis of variance and LSD test were carried out.

The adaptability and stability analyses were carried out using the methodology of Wricke [14], Eberhart and Russell [16], Lin and Binns [15], REML/BLUP [18], and GGE Biplot [17]. We considered a combination of seasons and locations when selecting environments.

The stability statistic of the Wricke methodology [14], called ecovalence (ϖ*_i_*), was estimated according to Equation (1):

(1) $$\varpi_{i}=\overset{n}{\underset{j-1}{\sum }}{\left(Y_{ij}-Y_{i}-Y_{j}+Y_{..}\right)}^{2}$$

where $Y_{ij}$ is the mean of genotypes *i* in environment *j*; $Y_{i}$ is the mean of genotypes *i* across all environments; $Y_{j}$ is the mean of environment *j* for all genotypes;$\;Y_{..}\;$ is the overall mean. Genotypes with low ϖ*_i_* values are considered stable and have smaller deviations from the environment.

The Eberhart and Russell method [16] was estimated according to Equation (2):

(2) $$Y_{ij}=\beta_{10i}+\beta_{i\;}I_{j}+\delta_{ij}+\varepsilon_{ij}$$

The hypotheses (H_0_:$\beta_{1i}$ = 1) and (H_0_:$\beta_{1i}+\beta_{2i\;}$) = 1 were evaluated by the test “t_α,m_”, where *α* is the significance level, and *m* is the degrees of freedom of the residual.

The method of Lin and Binns [15] is given by Equation (3):

(3) $$P_{i}=\overset{n}{\underset{j=1}{\sum }}\frac{{\left(X_{ij}-M_{j}\right)}^{2}}{2n}$$

where *P_i_* estimates the stability parameter of the genotype *i*; *X_ij_* is the yield of the *i-*^th^ genotype in the *j-*^th^ environment; *M_j_* is the maximum response observed among all genotypes in environment *j*; *n* is the number of environments.

In the REML/BLUP analysis [18], the statistical model for genetic evaluation was used according to the highest harmonic mean values of the genotypic values, according to Equation (4):

(4) Y=Xr+Zg+Wi+e

where Y is the data vector; r is the vector of block averages across environments (fixed) added to the overall average; g is the vector of genotypic effects (random); i is the vector of effects of the G × E interaction (random); and e is the vector of errors (random). X, Z and W represent the incidence of matrices for the effects mentioned above.

In the REML/BLUP analysis, selecting the highest harmonic mean of genotypic values (HMVG) implies the simultaneous selection of stability and grain yield. Adaptability refers to the relative performance of genotypic values (RPGV) across environments. Simultaneous selection of yield, stability, and adaptability can be performed by the harmonic mean of the relative performance of breeding values (HMRPVGi) method. Through this model, the genotypic values free of all interaction with environments were obtained by $\hat{\mu }+g_{i}$, where $\hat{\mu }$ is the average of all environments and provides a genotypic effect free from G × E interaction. For each j environment, the genotypic values (Vg) are predicted by: ${\hat{\mu }}_{j}+{\hat{g}}_{i}+{\left(\hat{g}e\right)}_{ij}$, where ${\hat{\mu }}_{j}$ is the mean of the j environment; ${\hat{g}}_{i}$ is the genotypic effect of i genotype in the j environment; and ${\left(\hat{g}e\right)}_{ij}$ is the effect of G × E interaction concerning i genotype. The prediction of genotypic values capitalizing the average interaction $\hat{g}e_{m}$ in the different environments is given by: ${\hat{\mu }}_{i}+{\hat{g}}_{i}+\hat{g}e_{m}$ and is calculated by:

$$\hat{\mu }+\frac{\left(\frac{\sigma_{g}^{2}+\sigma_{i}^{2}}{n}\right)}{\sigma_{g}^{2}}\;\hat{g_{i}},$$

where $\hat{\mu }$ is the overall average for all environments; n is the number of environments and $g_{i}$ is the genotypic effect of i genotype. The selection, considering the yield, stability simultaneously, and adaptability of safflower is given by Equation (5):

(5) $$HMRPGV_{i}=n\;/\;\frac{\sum_{j=1}^{n}\times 1}{Vg_{ij}}$$

The GGE biplot method [17] is given by Equation (6):

(6) $$Y_{ij}-y_{j}=y_{1}e_{i1}\varrho_{i1}+y_{2}e_{i2}\varrho_{i2}+e_{ij}$$

where $Y_{ij}\;$ represents the average trait of interest of genotype *i* in environment *j*; $y_{j}$ is the general average of the genotypes in environment *j*; $y_{1}e_{i1}\varrho_{i1}\;e\;y_{1}e_{i1}\varrho_{i1}$ are the first (PC1) and second (PC2) principal components, respectively; $e_{ij}$ is the error associated with the model of the *i-*^th^ genotype and *j-*^th^ environment.

The analyses conducting according to an LSD test, Wricke [14], Eberhart and Russell [16], Lin and Binns [15] were performed using the GENES^®^ software. Analysis by the GGE biplot method was performed using the R software with the *GGEbiplots* packages. The analysis by the method of mixed models was performed using model 54 (complete block design in several locations and one observation per plot) from the Selegen-REML/BLUP software [18].

## 3. Results and Discussion

The joint analysis of variance revealed a significant difference at 1% probability for genotype (G), environment (E), and the G × E interaction (Table 2). There is genetic variability for the traits, allowing for the selection of superior genotypes and exhibiting different performances according to the environment. The significance of the G × E interaction can affect the selection of safflower genotypes, making it difficult to recommend new cultivars. Several researchers also found significant interactions when studying safflower genotypes in other environments around the world: Iran [8,20,21,22], Turkey [23], and Brazil [5].

A significant variation of grain yield was observed between the four environments studied (Table 2). The environment represented by Campo Novo do Parecis-MT had the lowest average grain yield (278.71 kg ha^−1^), while in the Botucatu-SP(1), the highest average grain yield was obtained (1226.16 kg ha^−1^).

The grain yield of safflower genotypes varies due to the high dependence on genotypic and environmental conditions [21]. The significantly low grain yield averages observed in Campo Novo do Parecis-MT can be explained mainly by the high rainfall in the region (1940 mm per year). Safflower is a high-water-demanding crop that does not tolerate rain during the reproductive stages and can have its grain yield harmed by waterlogging [24,25]. Prolonged rains during flowering interfere with pollination and, consequently, seed formation [26].

Additionally, in Campo Novo do Parecis, the edaphic conditions of this environment indicate a soil with medium fertility, as shown in Table 1. Base saturations of 55.0% and 43.8 mmol_c_ dm³ of H + Al were observed. The high acidity combined with the low base saturation due to the predominance of iron and aluminum oxides promotes a high rate of phosphorus fixation in oxisols, resulting in low phosphorus availability for plants and consequently limiting crop yield [27,28,29].

The high grain yield of safflower in the “Botucatu-SP(1)” environment can be explained by some edaphic aspects. An elevated base saturation (94.0%) and pH close to 7.0 were observed. According to Pavlov and Tadorov [30], although safflower can be cultivated in a wide pH range, it adapts better to soils close to neutrality. The soil also contained high levels of calcium (98.0 mmol_c_ dm^−3^), magnesium (41.0 mmol_c_ dm^−3^), and phosphorus (38.0 mg dm^−3^). Well-nourished soils tend to increase safflower yield and oil content [25]. Several studies worldwide reported that P-rich soils showed better responses of yield components and grain yield in the safflower crop [31,32]. Especially when it comes to oxisols, which have a high phosphorus fixing capacity [28], another point is that in this environment, the lowest content of H+ Al (9.0 mg dm^−3^) was observed, allowing greater availability of phosphorus for plants, since aluminum oxide promotes phosphorus fixation in oxisols [28,29].

During the safflower growing season in Campo Novo do Parecis-MT, a large infestation of *Alternaria* spp was observed. All genotypes reacted similarly to the *Alternaria* spp. incidence. They showed a high susceptibility to the disease. No promising genotypes were found for resistance to *Alternaria* spp. Safflower grain yield can be reduced by up to 100%, depending on the intensity of the disease [33].

Although the tests carried out in Botucatu-SP were implemented in geographically close experimental areas, the Botucatu-SP(1) and Botucatu-SP(2) environments differ in several aspects that explain the difference in grain yield. First of all, the harvests were carried out in different years, which were characterized by different climatic conditions. Among the edaphic aspects, there was a great difference between the environments, as shown in Table 1, with an emphasis on low base saturation (32.0%), pH (4.4), and calcium content (15.0 mmol_c_ dm^−3^); and high H + Al (56.0 mmol_c_ dm^−3^) and presence of aluminum (1.0 mmol_c_ dm^−3^) in the “Botucatu-SP(2)” environment.

We observed a variation of only 3.48% for the oil content among the four environments (Table 2). Koutroubas et al. [34] and Omidi et al. [35] reported that this trait depends mainly on genetics, with less influence from the production environment of the plants.

Our results indicate that Campo Novo do Parecis-MT presented itself as a stressful environment for the plants, verified by the low yield of the crop; however, the highest average of oil content was registered in this location (Table 2). During stress, a change in plant dynamics can occur, leading to the higher translocation of photoassimilates to seeds. In stressful environments, changes in seed oil content were reported by Singh et al. [36] and Bortolheiro et al. [37].

The differences in grain yield and oil content between the genotypes (Table 3) are explained by the genetic constitution of the genotypes and by the genotype × environment interaction, which controls gene expression and, consequently, crop yield according to the environment [38,39].

Although the grain yield of all genotypes is statistically equal by the LSD test, the genotypes P43, P30, P28, P7, P9, P11, and P21 have a higher grain yield than IMA7326 (control), with the largest grain yield of P11 being about 64% higher than IMA7326 (Table 3).

The stability given by the Wricke method evaluates the trend of each genotype in different environments, considering the genotype with the lowest Wricke index to be stable (Wi%). For grain yield (Table 3), the lowest index was observed for P9 (0.41%), which is considered the most stable genotype, followed by P35 (1.29%) and P31 (1.98%). For oil content (Table 3), the Wricke method considered that the P35 genotype contributed more strongly to the G × E interaction (48.87%), while P7 and P11 genotypes were considered the most stable, at 0.43 and 1.23%, respectively.

According to Cruz et al. [13], the method proposed by Lin and Binns [15] does not present the disadvantages found using regression-based methods. It allows the identification of one or more genotypes with a performance close to maximum in the environments assessed through estimates of only one parameter (Pi). The most stable genotype has the lowest deviation from the maximum yield in each environment, the smallest Pi value. Thus, genotypes with lower Pi values respond more similarly to the ideal hypothetical genotype since they have a greater general adaptability. A superior genotype should have a high average yield and maintain it in all environments [40]. Thus, the P11 genotype stood out for presenting a performance closer to a hypothetical “ideal genotype” to grain yield and oil content (Table 3). This genotype had low Pi values and a satisfactory general mean, with a higher yield than the others. According to Oda et al. [41], the genotypes classified as more stable and adapted are generally among those with the highest yield when evaluated by the Lin and Binns method.

According to Eberhart and Russell [16], a stable genotype will be one with β = 1 and = 0. Genotypes with β > 1 could be better adapted to favorable growing conditions; those with β < 1 could be adaptable to environmental conditions that are not favorable, and those whose regression coefficients are equal to unity could show an average adaptation to all environmental conditions.

The stability and adaptability for safflower grain yield (Table 3), determined using the Eberhart and Russell methodology, indicated that the genotypes, IMA7326 and P30, were considered adapted to unfavorable environments. At the same time, P7 and P11 were deemed to be adapted to favorable environments.

The most favorable agronomical environment presents the lowest quadratic distance estimates between the real distance of the environments and the ideal environment [42]. Unfavorable environments are those with environmental factors difficult to control, such as low rainfall distribution, drought periods, insect attacks, diseases, etc. Such conditions limit the capacity of genotypes to express their potential.

P28, P35, P9, P11, P21, P31, and P14 were considered genotypes with broad adaptability. All genotypes were considered to have a low stability (S^2^d > 0), except P9 (S^2^d = 0.1). For the predictability of the behavior of genotypes in the environments, P7 (80.85%), P35 (86.10%), P31 (85.90%), and P9 (97.42%) were considered predictable genotypes. Based on the studied parameters, the P9 genotype had an average yield (726.91 kg ha^−1^) higher than the control, broad adaptation (favorable and unfavorable environments), good stability (S^2^d = 0.10), and high predictability (R^2^ = 97.42%).

In evaluating oil content, a t-test classified genotypes into four different categories (Table 3). The G7 genotype had high oil content; however, its adaptation is only to favorable environments (β1 = 1.34), with low stability (S^2^d = 14.60) and low predictability (R^2^ = 28.11). The genotypes P43, P30, P43, and IMA7326 showed adaptability to favorable environments, while for all others, no significance was verified in the adaptability analysis. As for behavioral stability, most genotypes were classified as non-stable, except for P31 (S^2^d = −0.05). As for the predictability of behavior, P7 and P14 were considered genotypes with a high predictability (94.82 and 99.89%, respectively).

Phenotypic stability is related to choosing the least affected genotypes by environmental variations. In contrast, adaptability identifies genotypes with predictable behaviors (predictability) that can adjust to the environmental variations or a definite subset of environments [43].

The advantages of using HMRPGVi include the selection for adaptability and genotypic stability since the effects of genotypes are considered to be random, the ability to work with unbalanced data and a heterogeneity of variances, and attention to results regarding the real magnitude of the evaluated traits, allowing its use in any number of environments [44].

For grain yield (Table 3), the P30 genotype obtained the best result (977.54 kg ha^−1^), and for oil content (Table 3), IMA7326 (27.48%) and P43 (24.25%) stood out, considered the most adapted and stable genotype.

The GGE biplot analysis of the genotype with environmental data addresses short-term applied questions and provides insights into long-term fundamental problems [45]. This method can analyze the G × E interaction and determine genotypes with a medium performance and high stability [46].

**Figure 1:**
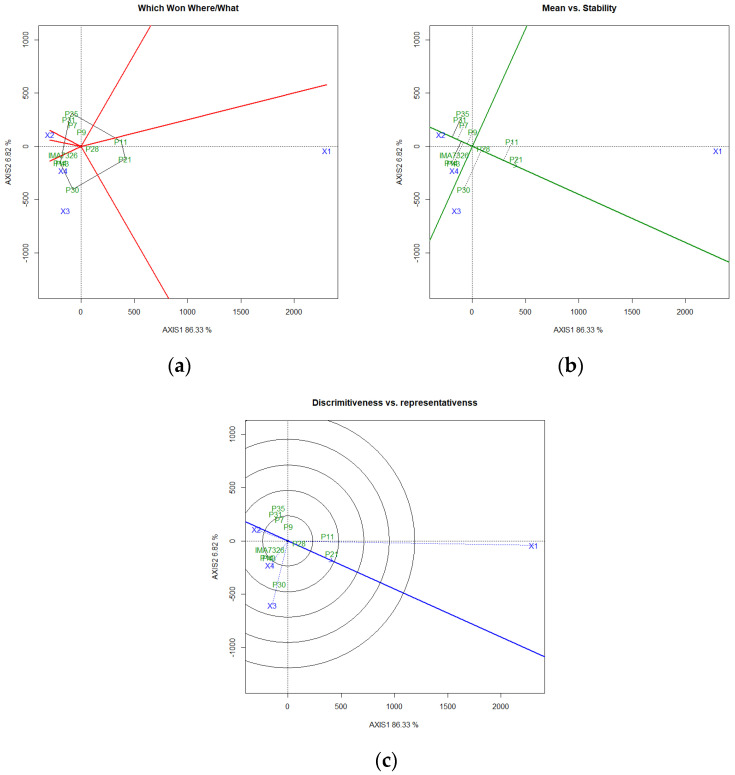
Polygon (**a**), stability (**b**), and ideal environment (**c**) tested for grain yield obtained by the GGE biplot method in safflower genotypes grown in four environments of the Brazilian Cerrado. Environments: X1: Bauru-SP; X2: Botucatu-SP(1); X3: Botucatu-SP(2); X4: Campo Novo do Parecis-MT.

In the graph, six sectors for grain yield were identified based on the vectors from the biplot center (0.0) of the GGE biplot analysis (Figure 1). Genotypes P28, P11, and P21 were grouped in the same sector, with a specific relationship with the Bauru-SP environment, genotypes, IMA7326, P43, P30, and P14, in the same sector as the environments, Botucatu-SP(2) and Campo Novo do Parecis-MT. The P7, P35, P9, and P31 genotypes were not positively associated with any studied environment (Figure 1a).

Grain yield and genotype stability were evaluated from the coordinate of the average environment (CAE). The ordered CAE is represented by two arrows, which point in the opposite direction from the origin of the biplot and indicate a greater effect of the G × E interaction and less stability, as well as separating the genotypes below the mean from the genotypes above the mean. P30, P28, and P21 were above the average grain yield. Based on the distance of these genotypes, the most stable were the P28 and P21 genotypes (Figure 1b).

The stability of growing environments can be seen in Figure 1c. Environments with a smaller vector of the biplot origin present greater yield stability, emphasizing the environments Botucatu-SP(1) and Campo Novo do Parecis-MT. Environmental vectors greater than 90° indicate a negative correlation between their responses; the genotypic responses tend to be divergent in these environments, as found in the Bauru-SP environment concerning the others.

**Figure 2:**
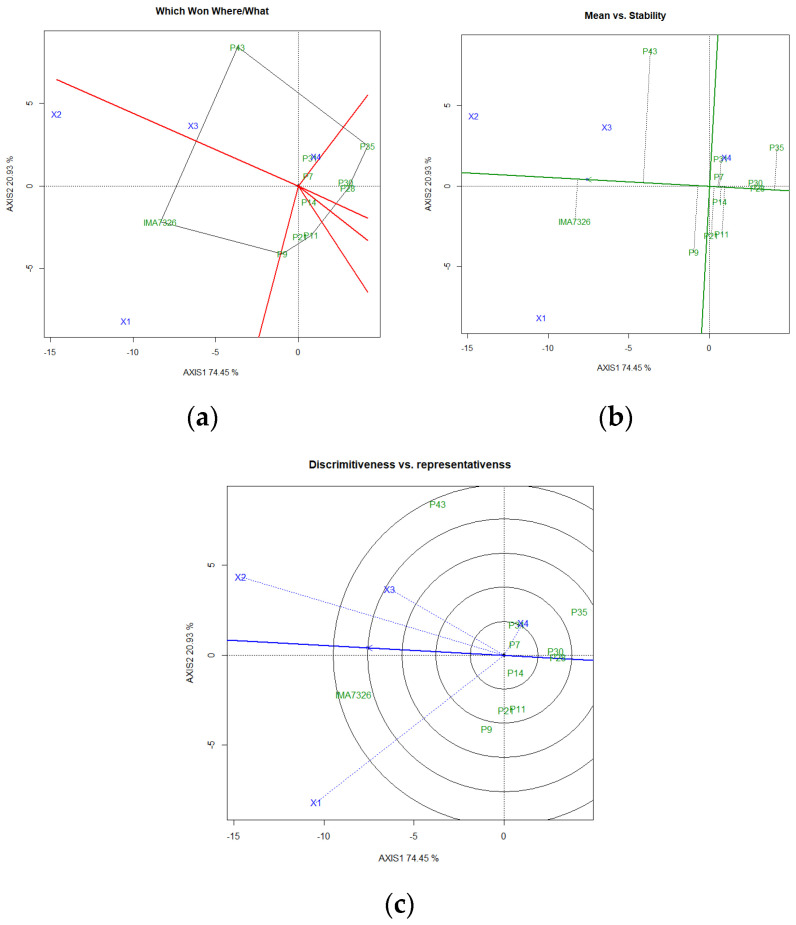
Polygon (**a**), stability (**b**), and ideal environment (**c**) tested for oil content obtained by the GGE biplot method in safflower genotypes grown in four environments of the Brazilian Cerrado. Environments: X1: Bauru-SP; X2: Botucatu-SP(1); X3: Botucatu-SP(2); X4: Campo Novo do Parecis-MT.

For the oil content by GGE biplot analysis, the formation of six sectors was also observed (Figure 2a). However, the grouping of genotypes and their relationship with the cultivation environments changed from that found for grain yield. The IMA7326 genotype was in the same sector as the Bauru-SP and Botucatu-SP(1), while the P43 and P31 genotypes were grouped in the same sector of Botucatu-SP(2) and Campo Novo do Parecis-MT. The other genotypes did not show associations with any studied environment. Considering all of the genotypes studied, P28, P30, and P7 were the most stable for oil accumulation (Figure 2b).

Concerning the environments, the smallest vector was found in Campo Novo do Parecis-MT; therefore, it was considered an environment of greater stability for the oil content. This environment was negatively associated with the Bauru-SP environment, being it contained the most contrasts for the ranking of genotypes concerning oil content (Figure 2c). In safflower cultivation, the response of different genotypes to environmental conditions should be considered, and stable genotypes should be chosen [47].

Therefore, based on all analyzes performed, the results evidenced the genotypes: P30, P35, P9, P11, and P31 as superior for grain yield and P43, P7, P11, and P31 for oil content.

Considering grain yield and oil content simultaneously, the genotypes P11 and P21 are recommended for the cultivation in this region since they produced 206.1 and 196.6 kg of oil per hectare, respectively.

Compared with other oilseeds of importance in Brazil, such as soybean, safflower does not compete directly by area since safflower cultivation occurs in the second harvest. Additionally, drought periods in the second harvest are common, and due to the drought tolerance of safflower, its cultivation is an alternative for corn producers.

## 4. Conclusions

In environments where rainfall coincides with the reproductive phase of safflower, grain yield is impaired, as observed in the experiment in Campo Novo do Parecis-MT. Environments with soil with high base saturation, high phosphorus, calcium, and magnesium levels, and pH close to neutrality (7.0) favor safflower grain yield, as observed in Botucatu-SP(1). Grain yield was more intensely affected by the genotype × environment interaction than oil content under the study conditions. The genotypes P11 (1045.6 kg ha^−1^, and 19.7%) and P21 (952.7 kg ha^−1^, and 20.6%) are recommended for cultivation in this region, considering their grain yield and oil content simultaneously.

Although studies on genotype × environment interaction in safflower are recent developments in Brazil, safflower is viable to use out of season in the Brazilian Cerrado. The crop can generate profits for farmers and be used for oil production in periods of uncertain corn production.

## Figures and Tables

**Table 1:** Details of safflower testing sites.

Characteristics	Environments			
	Bauru-SP	Botucatu-SP (1)	Botucatu-SP (2)	Campo Novo Do Parecis-MT
Growing season	2018	2018	2019	2019
Geographic region	Southeast	Southeast	Southeast	Central–west
State	São Paulo	São Paulo	São Paulo	Mato Grosso
Soil taxonomy (USDA)	Oxisol	Oxisol	Oxisol	Oxisol
Base saturation (%)	74.0	94.0	32.0	55.0
pH (H_2_O)	6.0	6.9	4.4	6.2
H + Al (mmolc dm^3^)	10.0	9.0	56.0	43.8
K (mmolc dm^3^)	1.0	2.0	2.0	1.6
Ca (mmolc dm^3^)	18.0	98.0	15.0	37.0
Mg (mmolc dm^3^)	9.0	41.0	9.0	13.0
Al (mmolc dm^3^)	0.0	0.0	1.0	0.0
P (mg dm^3^)	10.0	38.0	12.0	26.0
Organic matter (%)	1.1	0.0	3.0	3.0
Climate classification ^1^	Cwa	Cwa	Cwa	Aw
Annual average temperature (°C)	21.6	20.2	20.2	22.7
Accumulated annual rainfall (mm)	1170.0	1300.0	1300.0	1940.0
Altitude (m)	526.0	760.0	770.0	572.0

^1^ Cwa: Subtropical climate with dry winter; Aw: Humid tropical climate.

**Table 2:** Environment means and joint analysis of variance of safflower genotypes grown in four environments of the Brazilian Cerrado.

Environment	Grain Yield (kg ha^−1^)		Oil Content (%)	
	Average	CV (%)	Average	CV (%)
Bauru-SP	831.92	14.20	21.25	9.33
Botucatu-SP(1)	1226.16	17.71	19.91	10.46
Botucatu-SP(2)	621.81	18.19	18.85	8.60
C. N Parecis-MT	278.71	29.17	22.33	10.04
Average	739.65	19.82	20.58	9.61
			Mean square	
Source of variation		Df	Grain yield	Oil content
Blocks		2	1332.04	8.80
Genotype (G)		10	309,434.76 **	70.10 **
Environment (E)		3	5,186,528.38 **	76.55 **
G × E		30	528,892.39 **	25.57 **
Residue		80	20,126.31	3.98
MSr+/MSr−			7.13	1.91
CVg average (%)			20.99	11.40
CVe average (%)			19.18	9.70
CVg/CVe ratio			1.09	1.17

** Significant at *p* ≤ 0.01.

**Table 3:** Stability and adaptability of grain yield and oil content of safflower genotypes tested in four environments of the Brazilian Cerrado by the methodologies of Wricke, Lin and Binns, Eberhart and Russell, and HMRPGVi.

	Average		Wricke		Lin and Binns		Eberhart and Russel						HMRPGVi	
	GY	%O	GY	%O	GY	%O	GY			%O			GY	%O
Genotype	kg ha^−1^	%	Wi%	Wi%	Pi	Pi	β	S^2^d ^1/^	R^2^	β	S^2^d ^1/^	R^2^	kg ha^−1^	%
IMA7326	638.14 † a	26.42 † a	5.70	9.63	514,476.24	44.16	0.73 *	12.69 **	48.48	0.02 *	34.77 **	0.00	763.44	27.48
P43	639.91 a	23.40 a b	6.88	7.29	529,319.47	33.60	0.82	16.74 **	47.47	−1.34 **	14.60 **	28.11	693.56	24.25
P30	870.98 a	19.12 c	4.00	10.28	338,413.37	15.11	0.65 **	7.00 **	56.46	2.01*	5.10*	68.59	977.54	16.82
P28	764.14 a	18.43 c	2.82	7.32	207,086.42	15.65	1.00	6.78 **	75.83	1.38	3.08*	59.94	741.44	16.65
P7	754.77 a	19.92 b c	4.13	0.43	367,791.77	11.15	1.28 *	8.43 **	80.85	0.71	−1.23	94.82	761.28	19.30
P35	543.13 a	17.77 c	1.29	48.87	470,723.28	45.23	0.93	2.64 **	86.10	2.01 *	10.32 **	54.68	493.35	15.70
P9	726.91 a	20.78 b c	0.41	5.11	290,372.29	23.02	1.11	0.10	97.42	1.15	7.65 **	33.75	695.76	19.96
P11	1045.63 a	19.71 b c	26.33	1.23	55,876.87	5.69	1.54 **	62.12 **	47.06	0.89	4.53 *	31.94	695.78	18.51
P21	952.72 a	20.64 b c	37.26	4.42	117,960.49	9.89	0.87	97.49 **	15.53	1.60	1.77	74.35	566.32	19.09
P31	563.06 a	19.94 b c	1.98	3.69	485,696.85	11.09	1.12	4.20 **	85.90	0.78	−0.05	62.19	825.59	19.27
P14	636.95 a	20.27 b c	9.19	1.72	565,202.05	13.48	0.95	23.58 **	46.52	1.80	−1.32	99.89	559.88	18.79

^1/^ Divided by 10,000; † different letters indicate significant differences, according to the LSD test. (*p* < 0.05); **: 1% significant; *: 5% significant.

## Data Availability

The data presented in this study are available on request from the corresponding author.
